# Coronary Artery Bypass Grafting in Acute Ischemic Heart Failure: Where do We Stand? (And Where Should We Go?)

**DOI:** 10.21470/1678-9741-2018-0329

**Published:** 2018

**Authors:** Fernando B. Kubrusly, Paulo André Bispo Machado-Junior

**Affiliations:** 1 Incor Curitiba, Instituto Denton Cooley, Hospital do Coração de Curitiba, Curitiba, PR, Brazil.; 2 Escola de Medicina, Pontifícia Universidade Católica do Paraná (PUCPR), Curitiba, PR, Brazil.

Acute heart failure (AHF) refers to rapid onset or worsening of signs and symptoms of heart failure, often requiring hospitalization and urgent treatment, due to its potentially life-threatening condition^[1]^. The vast-majority of patients that suffers from heart failure has acute worsening episodes that become more frequent as the disease progresses.

More than half of these patients are known to have an ischemic etiology, and early revascularization improve clinical outcomes^[2,3]^, and the immediate invasive strategy with intent to perform revascularization in patients with both AHF and acute coronary syndrome is recommended in guidelines^[4,5]^. However, the choices of revascularization are still controversial subject: either percutaneous coronary intervention (PCI) or coronary artery bypass grafting (CABG).

Which one is better has been long debated. Trials comparing both techniques have shown that the rates of most adverse clinical outcomes favor CABG, which turns this technique as the preferred revascularization strategy in patients with multivessel disease. Unfortunately, most of the trials comparing PCI versus CABG were designed in the setting of stable coronary artery disease. The majority of patients included in these studies had two vessel disease and preserved left ventricular function, differing from the patients with AHF. Even so, there’s still no evidence of survival improvement when PCI was compared to CABG, especially in the long-term comparison.

A recent multicenter prospective cohort study, The Korean Acute Heart Failure Registry, evaluated 5625 patients enrolled prospectively from March 2011 to February 2014^[6]^. In this analysis, 717 patients received CABG or PCI during the hospitalization for AHF. Adverse outcomes (death, ischemic stroke and a composite outcome of death and readmission for heart failure worsening or cardiovascular causes) were used for propensity score matching. The authors concluded that, compared to PCI, CABG was associated with significant lower all-cause mortality in patients with AHF. The rate of death from any cause over 4 years was lowered by 40% among patients who underwent CABG than those who received PCI. These are very impressive results.

Another recent multicenter observational analysis between 2004 and 2014 among 7 medical centers, reporting to the Northern New England Cardiovascular Disease Study group, evaluated more than 70,000 patients who underwent CABG (n=18,292) versus PCI (n=55,438)^[7]^. After applying inclusion and exclusion criteria from the Surgical Treatment for Ischemic Heart Failure trial, the study showed that CABG was associated with improved long-term survival when compared to PCI in patients with ejection ≤ 35% and 2- or 3-vessel disease. In the long-term analysis, CABG was associated with lower incidence of all-cause mortality, being CABG strongly considered in these patients with ischemic cardiomyopathy and multivessel coronary disease.

The better results for surgery might be attributable to the ability to achieve complete revascularization in extensive coronary artery disease^[8]^. The complete revascularization rate, defined as all stenotic main-branch vessels being treated^[9]^, is always significantly higher in the CABG group than in the PCI group in most studies.

PCI is a well-established strategy and the advance and progress of the stents is beyond question. However, for patients that are experiencing signs and symptoms of heart failure, surgery is still the best and safer option. Besides, after 50 years the surgical field has also innovated and advanced. The use of multiple arterial grafts that have superior long-term patency has significantly improved the survival rates. Also, off-pump surgery has reduced aortic manipulation and consequently the rates of stroke and heart failure after surgery on specific patients.

It is unquestionable and a very widely known (but unfortunately not always remembered) fact that CABG yields better results and therefore a better and longer life ahead for these patients.
